# Research hotspots and trends in pediatric telemedicine: a CiteSpace-based bibliometric analysis (1978–2025)

**DOI:** 10.3389/fped.2025.1590663

**Published:** 2025-07-30

**Authors:** Hong Xie, Dan Luo, Bingyao Kang

**Affiliations:** ^1^ Department of Pediatric Outpatient Nursing, West China Second University Hospital, Sichuan University, Chengdu, Sichuan, China; ^2^ Key Laboratory of Birth Defects and Related Diseases of Women and Children Sichuan University, Ministry of Education, Chengdu, China; ^3^ West China Nursing School, Sichuan University, Chengdu, Sichuan, China

**Keywords:** telemedicine, pediatric, bibliometric analysis, data visualization, citespace

## Abstract

**Aim:**

Given the rapid growth yet fragmented nature of pediatric telemedicine research, this study employed bibliometric analysis to address specific research questions regarding key publications, leading authors, core journals, prominent institutions, thematic structures, and research theme evolution. The findings were aimed at providing an objective, panoramic view to guide future research priorities and clinical practice development in pediatric telemedicine.

**Methods:**

A retrospective bibliometric analysis was conducted on publications in the field of telemedicine in pediatrics, covering the time period from 1978 to 2025. The data were downloaded from the Web of Science Core Collection on March 4, 2025. This study analyzed the annual number of publications and citations, explored relationships between authors, institutions, countries, and keywords, and identified emerging trends and research frontiers in the field of telemedicine in pediatrics.

**Results:**

A total of 114 countries and 120 institutions published articles on pediatric telemedicine. Co-occurring keywords and cluster analysis indicate that recent major research trends in the field of pediatric telemedicine include telepsychiatry for mental health, autism spectrum disorders, and parent training, as well as digital health for care, service, and quality of life. Additionally, tele-diagnosis and care of, obesity, retinopathy of prematurity, critical care, and traumatic brain injury are also popular research areas.

**Conclusion:**

The United States has a significant presence in the field of pediatric telemedicine. Although telemedicine is growing rapidly in pediatrics, it cannot replace face-to-face care.

## 1 Introduction

Pediatricians are the primary healthcare providers for children. Currently, there is a global shortage of pediatricians, with only 0.05–1.6 pediatricians per 1,000 children in Europe (1). In developing countries such as China, there is a severe shortage of pediatricians, with geographic and economic disparities leading to an uneven distribution of pediatricians. Low-income rural areas are in greater need of pediatricians to provide access to healthcare for children (2). The lack of specialised pediatricians and remote locations pose significant barriers to children's access to healthcare services (2, 3). However, the use of pediatric telemedicine presents an opportunity to provide pediatric healthcare services in remote areas and low-income countries (4).

Telemedicine, defined as “treatment at a distance” by the World Health Organization, refers to healthcare professionals' use of information and communication technologies to exchange medical information across locations (5). This innovative approach has revolutionized medical practice through its capacity to improve healthcare accessibility, enhance treatment outcomes, and enable remote patient monitoring (6). The technology finds particular relevance in pediatric care, supporting applications ranging from chronic disease management and emergency medicine to home healthcare and diagnostic assistance (7–9). Its dual benefit system creates value for both patients and providers: children receive care in home environments through mobile monitoring devices and virtual consultations, reducing hospital readmissions and family expenses, while clinicians can remotely assess patients, review medical imaging, and coordinate hospital-based treatments without physical displacement (10). This contactless care model additionally mitigates infection risks by minimizing direct interactions between healthcare workers and patients (11).

The field of pediatric telehealth is evolving rapidly, making it hard for most to keep up. While meta-analyses on telehealth applications in pediatrics have been conducted, they mainly serve as theoretical expansion tools to explain outcomes without exploring underlying processes (7, 12). In contrast, bibliometric analysis evaluates social and structural relationships among research components like authors, countries, institutions, and topics, summarizing the bibliometric and intellectual structure of a specific field (13), and thus may provide a decision-making basis for telehealth technology developers and pediatric clinical practitioners. However, despite increasing interest in pediatric telemedicine applications, bibliometric analyses in this area are relatively scarce, possibly due to the lack of in-depth exploration of long-term efficacy data and personalized intervention protocols in existing literature. Given these limitations, research questions should center on using bibliometric methods to comprehensively reveal the current state, trends, and challenges in the field, providing a scientific foundation for targeted interventions. This study aimed to identify key publications, authors, journals, institutions, thematic structures, research evolution, and emerging trends, offering an objective overview and insights to guide future research and clinical practice.

## 2 Method

### 2.1 Data sources

CiteSpace is a visual analytic tool for analyzing trends and patterns in the scholarly literature of a field of research (14). The data were downloaded from the Web of Science Core Collection (WoSCC) on March 4, 2025. The WoSCC is a widespread and significant citation database utilized to chart scientific knowledge and technology. It is an efficient source of data retrieval for scientific metrological analysis. The retrieval strategy utilized topic retrieval and Boolean logical operators to construct retrieval expressions: (TS = telemedicine OR telehealth OR “remote consultation” OR “remote diagnosis” OR “remote clinic” OR “remote surgery” OR “remote pathology” OR “remote ECG” OR “remote ultrasound” OR “remote rehabilitation” OR “remote nursing” OR “remote health care” OR “Internet medical services” OR “Internet hospital”) AND TS = (“Child” OR “children” OR “pediatric” OR “youth” OR “school age” OR “adolescent” OR “preschool” OR “primary school” OR “middle school” OR “senior high school” OR “toddler” OR “infant”). The publication time ranged from inception to March 4, 2025. A total of 6,276 articles were retrieved.

### 2.2 Study inclusion/exclusion criteria

**Figure 1:**
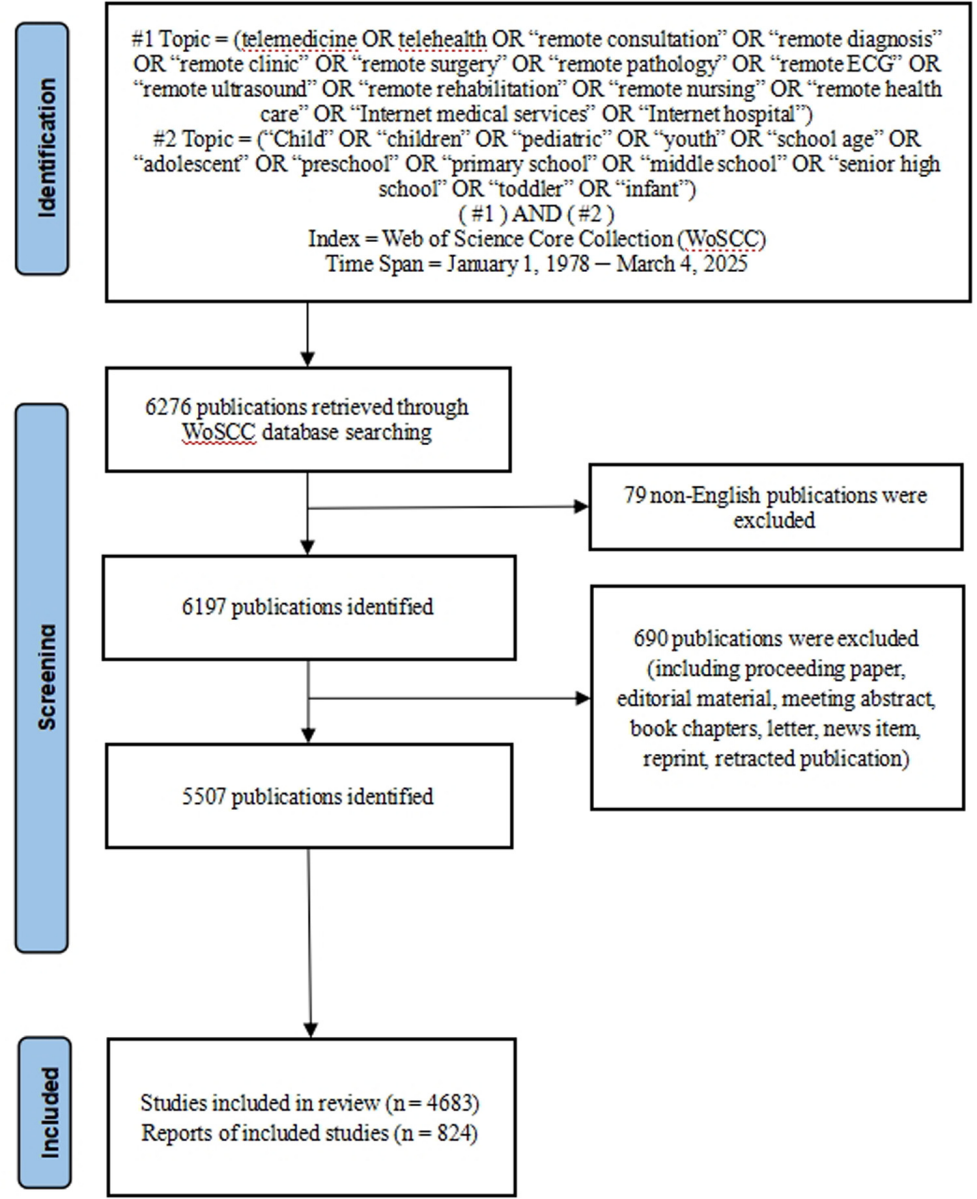
Flow chart of literature screening included in this study.

Study inclusion criteria were literature related to the telemedicine in pediatric, original research or review type of articles, and English language publications. Study exclusion criteria included literature such as meeting abstracts, editorial material, letters, news items, and duplicates. Two researchers read the titles and abstracts independently and removed literature not related to the mental health of nursing students. After the screening process, 5,507 publications were finally included in the analysis, as shown in Figure 1.

### 2.3 Data analysis

CiteSpace was analyzed the annual number of publications and citations, explored relationships between authors, institutions, countries, and keywords, and identified emerging trends and research frontiers in the field of telemedicine in pediatrics. Additionally, keywords with strong citation bursts over time were captured. There are various nodes and links present in CiteSpace visualization knowledge graphs. Hot spots or turning points in this domain are usually identified as nodes with high centrality. First, we downloaded all of the literature retrieved by WoSCC and then exported the full record, including cited references, in Refworks format. We saved this information in a text format to establish a database. Finally, we used CiteSpace 6.1.R6 software to deduplicate and visualize the included literature. The collaboration and document co-citation analyses in CiteSpace were configured with a temporal segmentation of 1978–2025 (48 annual slices, 1-year intervals), a node selection threshold of Top 50 items per slice (retaining the 50 most cited/frequent entries annually), and network pruning via Pathfinder scaling applied to sliced subnetworks. Cluster analysis was conducted using CiteSpace to reveal the main topics based on co-occurrence keywords. To evaluate the clusters, the silhouette function was utilized. A significant partition structure is indicated by a Modularity value (Q) over 0.3, and the clustering results are considered convincing when the Silhouette value (S) is over 0.7 (15).

### 2.4 Design and ethics

This retrospective bibliometric analysis mainly examined published articles and did not involve any human clinical trial studies. Consequently, ethical approval was not required from the relevant ethics review committee.

## 3 Results and discussion

### 3.1 Annual publication trend

**Figure 2:**
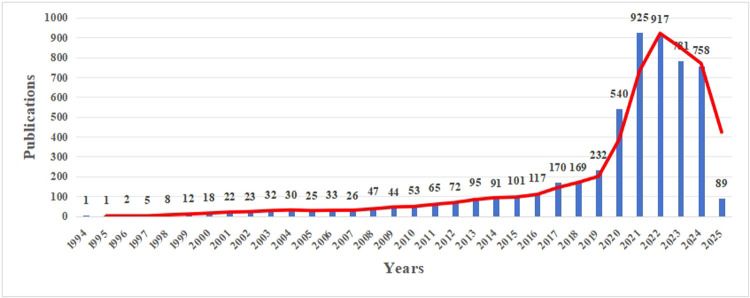
Year-wise publication of bibliometric papers from inception to March 4, 2025.

To establish temporal benchmarks for field evolution, we analyzed publication trends (Figure 2). Prior to the pandemic, the number of publications on this topic demonstrated steady growth, with an average annual growth rate of approximately 24.21% from 2015 to 2019. This indicated a growing interest and gradual development in the application of telemedicine within pediatric care. However, the onset of the COVID-19 pandemic in 2020 marked a dramatic surge in publications, with a growth rate of 132.76%, followed by a continued substantial increase in 2021. This study highlighted the pivotal role telemedicine had played during the pandemic, having become an essential tool for delivering healthcare services while minimizing viral transmission risks (16, 17).

Post 2022, a decline in publication numbers was observed, which could be attributed to several factors. These included a potential shift in research focus to other pressing areas, practical challenges encountered in implementing telemedicine, the maturation of existing technologies that led to a focus on applied research, changes in funding and policy support, and a general “return to normalcy” in research priorities. Despite the decrease, the number of publications remained higher than pre-pandemic levels, indicating that sustained interest in telemedicine within pediatric care had persisted. The initial rapid adoption during the pandemic had paved the way for further exploration of its long-term potential to enhance access, efficiency, and patient outcomes in pediatric healthcare.

### 3.2 Author, country and institutional cooperation network

To pinpoint dominant contributors, co-authorship networks were constructed to map institutional and country relationships. Our analysis revealed that 114 countries and 120 institutions had published research on pediatric telemedicine between 1978 and 2025. The top 10 countries and institutions were identified (Table 1). The results demonstrated that the United States had published the largest number of articles (2,985), accounting for 54.20% of the total papers. This indicated that the United States had held a dominant position in the field of pediatric telemedicine.

**Table 1:** Top 10 countries, institutions, authors and keyword.

Rank	Country	*N*	Institution	*N*	Authors	Count of articles	Year of the first article	Keywords	*N*	Centrality	Keywords
1	USA	2,985	Harvard Med Sch	133	Marcin, James P	52	2008	children	1,168	0.14	children
2	AUSTRALIA	441	Univ Washington	125	Smith, Anthony C	32	2007	telemedicine	906	0.11	prevalence
3	CANADA	369	Univ Queensland	122	Ray, Kristin N	28	2017	care	657	0.10	program
4	ENGLAND	272	Univ Calif Davis	114	Wade, Shari L	16	2009	telehealth	553	0.09	health
5	ITALY	214	Univ Penn	109	Chuo, John	13	2018	adolescent	360	0.09	outcome
6	INDIA	128	Childrens Hosp Philadelphia	97	Armfield, Nigel R	13	2010	intervention	359	0.08	telemedicine
7	GERMANY	109	Boston Childrens Hosp	97	Myers, Kathleen	12	2013	impact	333	0.08	care
8	PEOPLES R CHINA	90	Univ Colorado	96	Nelson, Eve-Lynn	11	2011	health	306	0.08	adolescent
9	BRAZIL	90	Univ Cincinnati	87	Mcconnochie, Kenneth M	11	2006	management	285	0.08	service
10	SPAIN	82	Univ Toronto	85	Dharmar, Madan	11	2009	outcome	277	0.08	satisfaction

**Figure 3:**
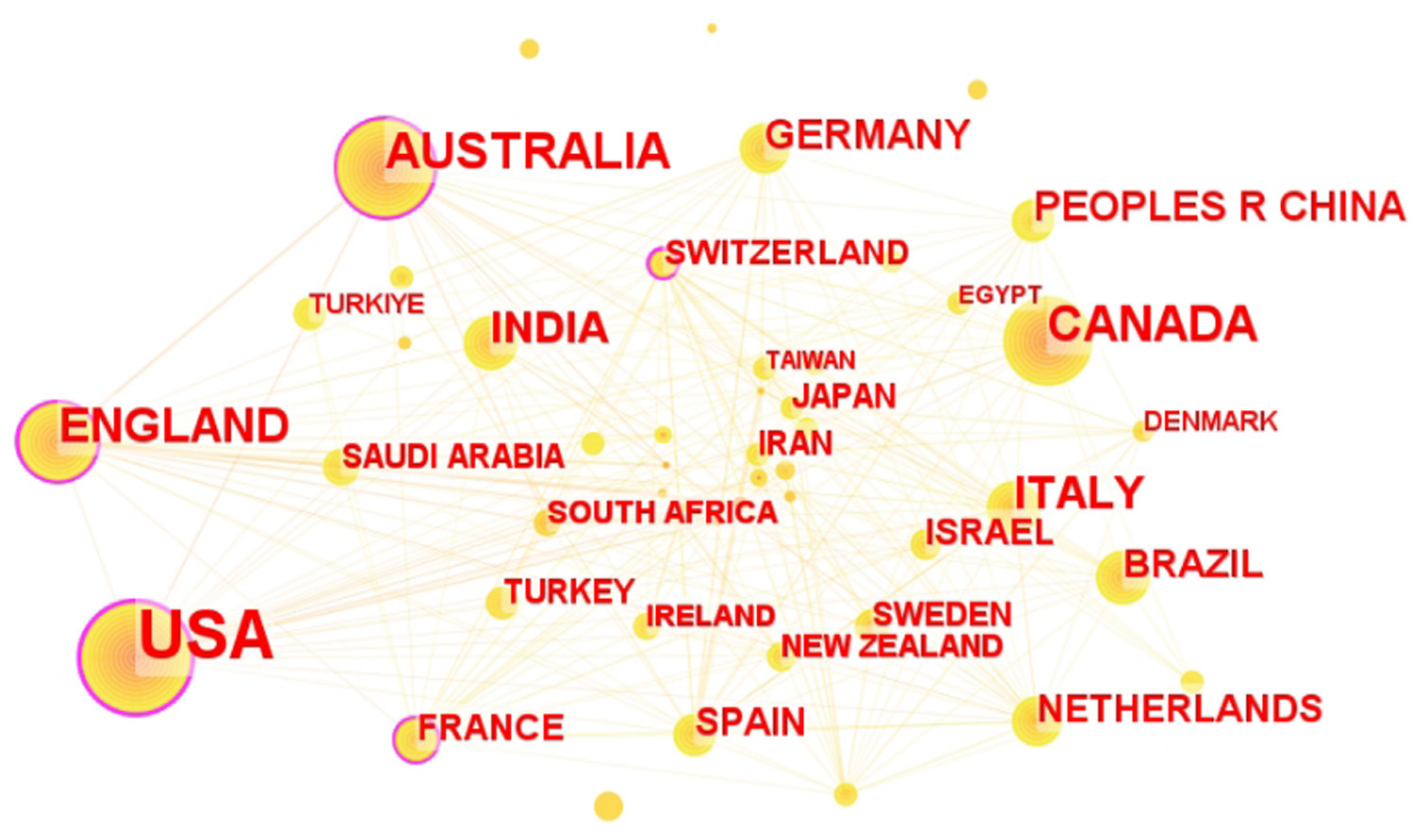
Network diagram in the field of pediatric telemedicine. The size of node reflects the co-occurrence frequencies, and the links indicate the co-occurrence relationships. The colour of node and line represent different years, and node with purple round means high betweenness centrality (>0.1).

**Figure 4:**
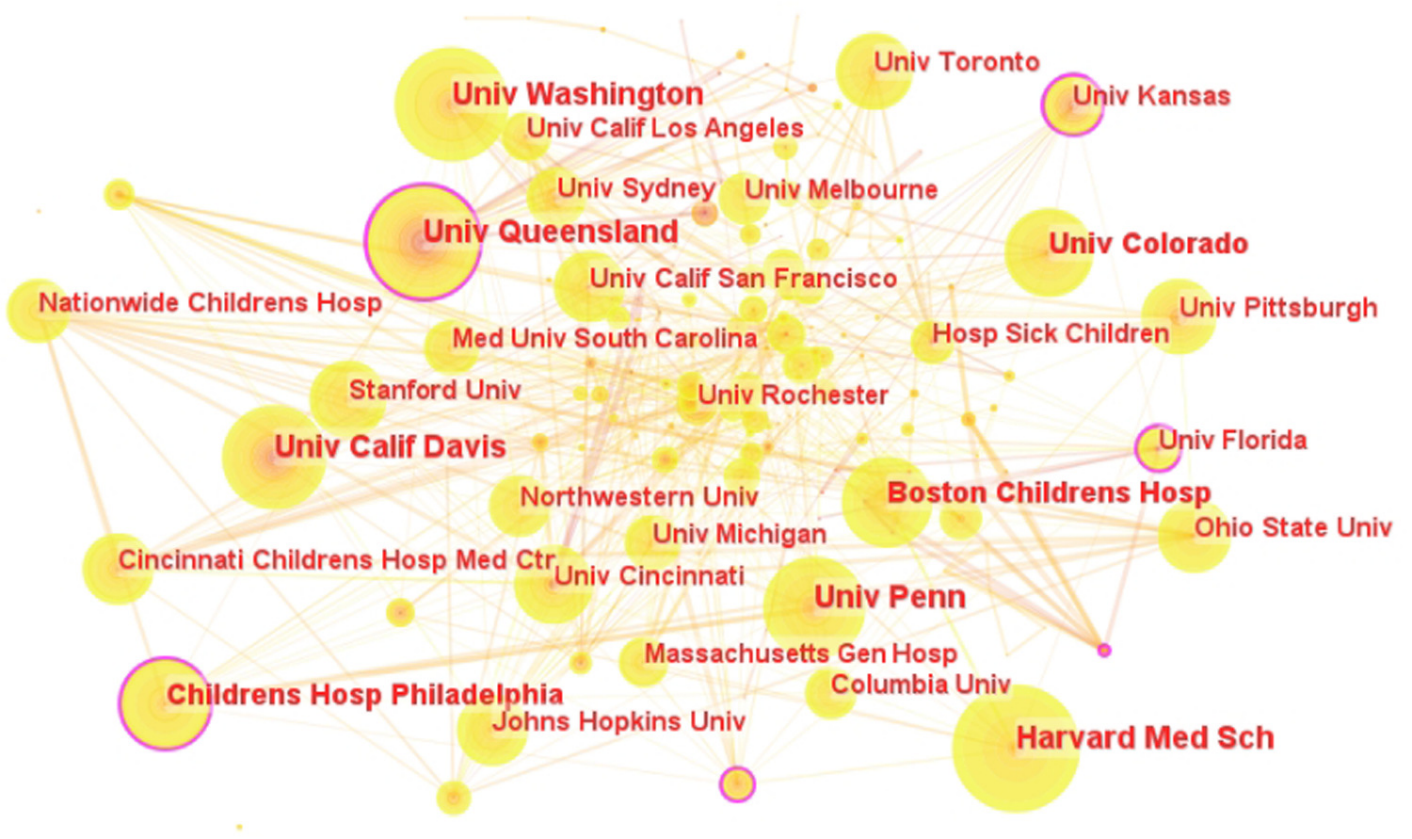
Institutional cooperation network in the field of pediatric telemedicine. The circular nodes represent institutions; the links between nodes represent interactions between institutions.

Cooperation network analysis was central to pinpointing the most productive and collaborative authors, institutions, and countries driving pediatric telemedicine research. The statistical results and CiteSpace analysis revealed that the majority of the top ten publishing institutions were located in the United States, highlighting the country's dominance in this field (Figures 3, 4). Harvard Medical School, located in the USA, was ranked as the top institution with 133 publications (Table 1). Regarding their partnerships, it was evident that institutions with the most publications had established strong connections with each other. The University of Florida exhibited the highest centrality (0.19), followed by the University of Queensland (0.16) and the Children's Hospital of Philadelphia (0.12). The research showed extensive collaboration between institutions and countries, particularly involving the United States, England, and Australia. Researchers could select relevant institutions for collaboration using the institutional cooperation network.

Through analysis of the number of papers published by authors and their collaborative networks, we found that eight of the top ten authors researching pediatric telemedicine were from the United States (Table 1). This further illustrated the leading position of U.S. academics in this field. At the top of the list was James P. Marcin from the University of California Davis, with 52 publications. His research focused on pediatric emergency telemedicine, pediatric intensive care unit telemedicine, and telemedicine applications for rural health issues.

**Figure 5:**
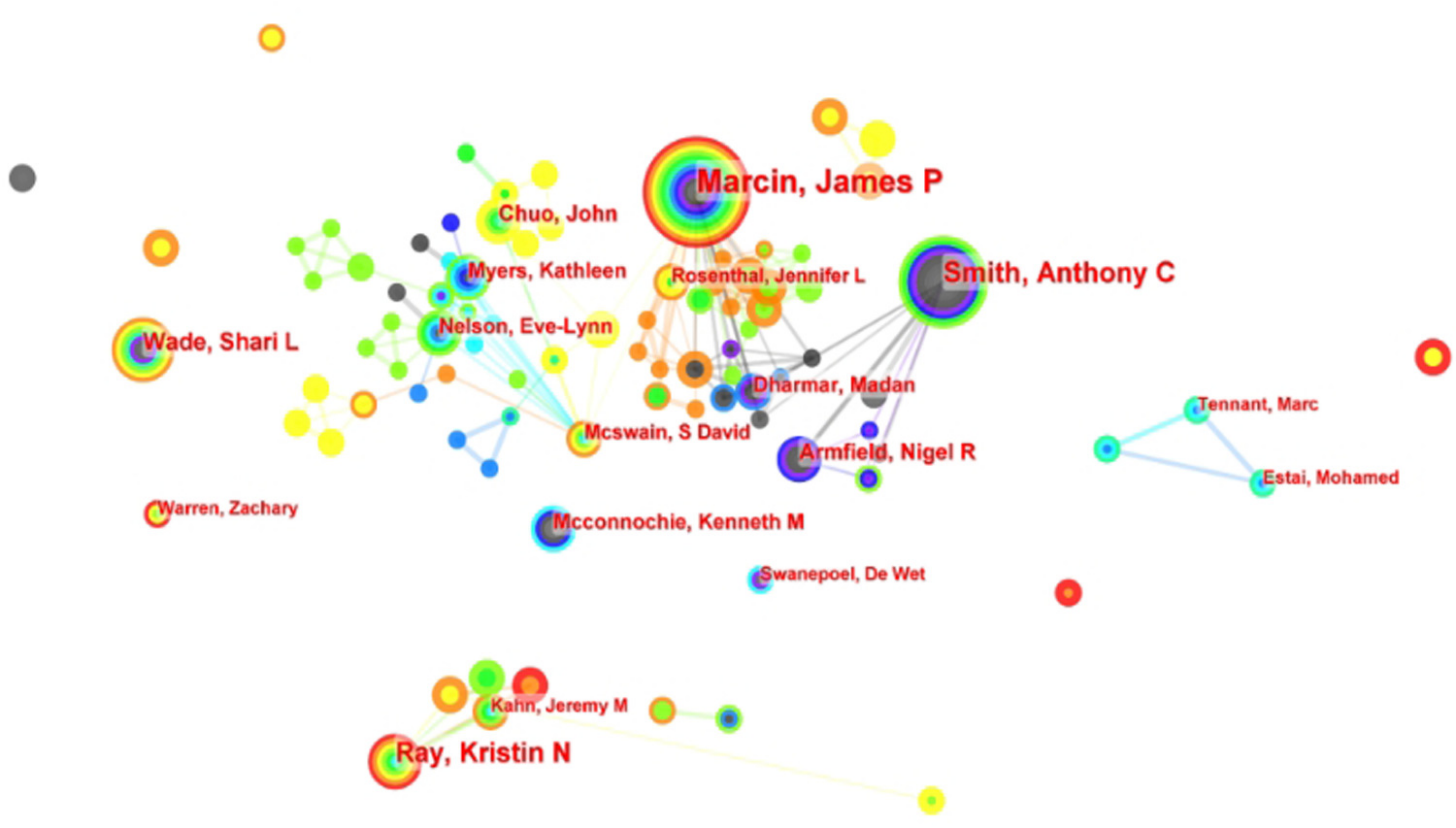
Visualization and analysis of co-authors in publications related to pediatric telemedicine. Circle nodes represent the author of papers; links between nodes represent partnerships.

Analysis of the collaborative relationships between scholars revealed that these relationships were distributed in a decentralized manner (Figure 5). While some academics had close collaborative ties, it was notable that they all came from the same research organization. For instance, Marcin, James P.; Dharmar, Madan; and Rosenthal, Jennifer L shared close collaborative relationships, all being affiliated with the University of California Davis.

### 3.4 Literature from the last three years

To gain a comprehensive understanding of research trends in pediatric telemedicine, a review of the literature over the past three years revealed that the three most frequently cited areas were health literacy, autism spectrum disorders (ASD), and psychology. Health literacy had emerged as a prominent focus in pediatric telemedicine research, particularly as the COVID-19 pandemic drove the rapid adoption of telemedicine as the primary means of patient-provider interaction. Telemedicine technology provided a platform for children to learn and improve their health literacy, enabling them to acquire basic health knowledge and skills through digital tools (18). Furthermore, telemedicine was being utilized in child and adolescent psychiatry to diagnose and intervene in neuropsychiatric disorders such as autism (19). In parallel, as societal awareness of children's mental health issues grew, tele-technology was increasingly employed in psychological counseling and treatment (20).

### 3.5 Analysis of hotspot evolution

**Figure 6:**
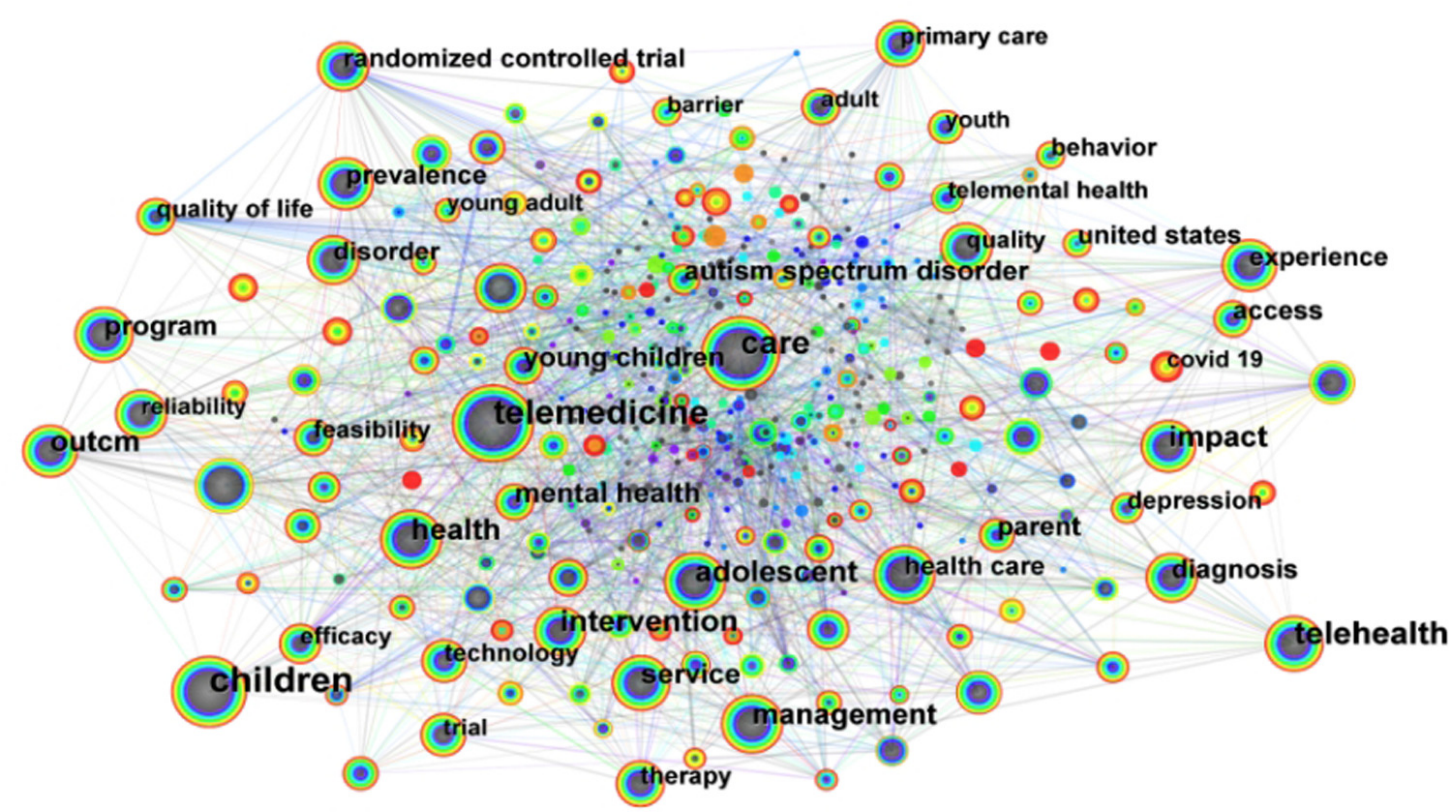
Citespace visualization of keywords involved in the research of pediatric telemedicine—network of the main keywords in publications.

Keywords represented the core content of publications. Analyzing their co-occurrence revealed the main research topics and the conceptual structure of the pediatric telemedicine field by identifying clusters of closely related terms. The nodes in Figure 6 represented corresponding keywords, with node size indicating the number of articles in the field that contained these keywords. The study identified the top 10 high-frequency and high-centrality keywords (Table 1). Centrality served as a critical indicator for gauging the significance of nodes depicted in the graph. High-centrality keywords demonstrated the prominence of relevant research in pediatric telemedicine. The high-centrality keywords included children (0.14), prevalence (0.11), and program (0.10).

**Figure 7:**
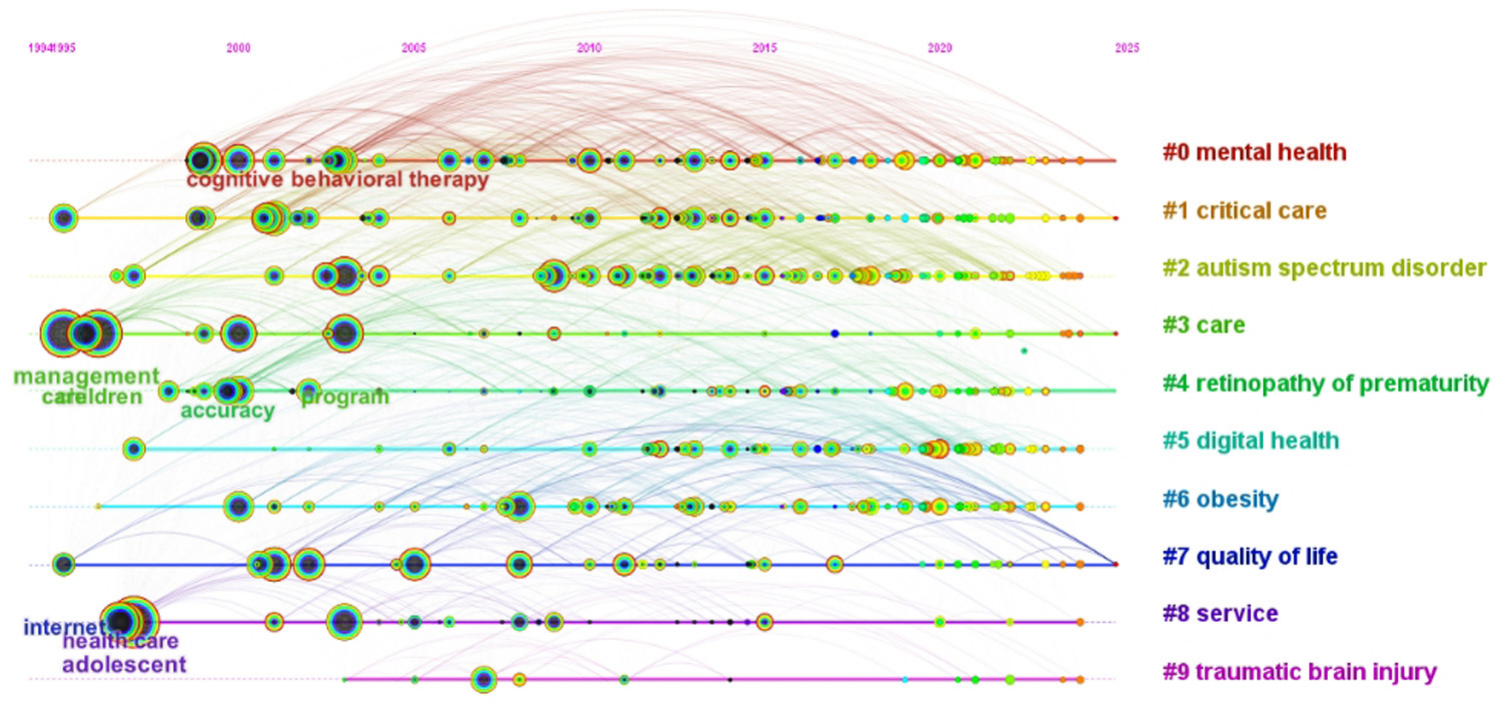
Keywords clustering analysis timeline map of research on pediatric telemedicine.

To track thematic shifts, timeline visualization traced cluster development. We performed cluster analysis on co-occurring keywords to group related research topics and visualized the evolution of keyword clusters over time. The articles in pediatric telemedicine were arranged in a timeline manner using a clustering network, as shown in Figure 7. Noun phrases were extracted from the titles and summaries of the cited articles to identify thematic patterns for each cluster, and the most representative noun phrases were used to label the clusters. Co-occurring keywords were divided into ten sub-clusters (Table 2). The Modularity value (Q = 0.4015) indicated a significant partition structure, and the Silhouette value (S = 0.6956) confirmed convincing clustering results (15). This visualization tracked the birth, development, maturity, and potential decline of research themes, revealing the dynamics of the pediatric telemedicine field's intellectual structure. Co-occurring keywords and cluster analysis indicated that recent major research trends in pediatric telemedicine included mental health, autism spectrum disorder, and digital health for care, service, and quality of life. Additionally, tele-diagnosis and care for obesity, retinopathy of prematurity, critical care, and traumatic brain injury were also identified as popular research areas.

**Table 2:** Keyword cluster analysis .

Label (LLR)	Silhouette	Included keywords	Mean (Year)
#0 mental health	0.68	mental health; telemental health; psychiatric problems; anxiety; depression	2013
#1 critical care	0.665	critical care; pediatric palliative care; home monitoring; access to care; NICU	2012
#2 autism spectrum disorder	0.678	autism spectrum disorder; telemedicine; parent training; autism; telepractice	2016
#3 care	0.77	care; management; asthma; parent training; developing countries	2005
#4 retinopathy of prematurity	0.686	retinopathy of prematurity; telemedicine; screening; perimetry; strategy	2011
#5 digital health	0.633	digital health; pre-exposure prophylaxis; primary health care; remote consultation; mobile application	2017
#6 obesity	0.612	obesity; type 1 diabetes; exercise; continuous glucose monitoring; pediatric obesity	2015
#7 quality of life	0.812	quality of life; children; telehealth; intervention; sleep	2014
#8 service	0.740	service; health care; satisfaction; consultation; reliability	2006
#9 traumatic brain injury	0.924	traumatic brain injury; behavioral interventions; intervention; telerehabilitation; exposure-based group	2013

Mental health covered a broad spectrum of mental, emotional, social, and behavioural functioning and existed on a continuum from positive to negative (21). Around two-fifths of children and adolescents under the age of 18 in the United States were reported to meet criteria for mental disorders (22). The provision of mental health services to children and adolescents in the United States was deemed inadequate, particularly in rural or underserved communities. Additionally, a shortage of child and adolescent psychiatrists resulted in the majority of children with behavioural health problems receiving no treatment (23, 24).

The implementation of telemedicine in mental health services removed financial and time barriers for patients. Providing psychological counselling services remotely helped to reduce the stigma attached to patients with mental disorders and increased the rate of regular attendance at clinics (23). Telepsychiatry for children and young people was identified as a viable, acceptable, and sustainable approach that could be applied in schools, juvenile detention centres, families, and clinical settings (25). While telepsychological interventions had been shown to be effective, several barriers to their implementation were noted, including technical difficulties, privacy and security concerns, and ethical issues (26). However, the American Telemedicine Association developed a practice guideline for telemedicine in child and adolescent mental health and behavioural services to ensure appropriate patient care (27).

The increasing prevalence of ASD, which had reached 1 in 36 children aged 8, had driven a surge in demand for accessible and effective healthcare services (28). This demand, coupled with the inherent challenges of accessing specialized care for ASD, had fueled the rapid growth of telemedicine in this field. Unlike episodic pediatric conditions (e.g., acute infections), ASD demanded intensive, longitudinal interventions that strained traditional healthcare systems, particularly in under-resourced regions lacking developmental specialists. Telemedicine addressed this gap through three synergistic drivers: (1) caregiver-mediated scalability, enabling remote training of parents to implement evidence-based behavioral strategies; (2) diagnostic adaptability, as ASD assessments relied on observational tools (e.g., social interaction analyses) rather than physical exams, making video-based platforms uniquely effective; and (3) geographic urgency, with over 50% of U.S. counties lacking ASD specialists, necessitating remote solutions to prevent delays in critical early interventions (29, 30).

Telemedicine was increasingly being used in pediatric care, covering a wide range of scenarios from chronic disease management to mental health support.7 For example, in the case of type 1 diabetes, telemedicine had significantly improved treatment adherence and quality of life by reducing the travel burden on patients and their families (31). This trend was similar to the application of telemedicine in other medical fields, such as ophthalmology, where remote screening and chronic disease monitoring had significantly improved patient accessibility and diagnostic efficiency (32). However, the uniqueness of pediatric telemedicine lay in its strong focus on the psychological needs and social support of children and their families, which differed from the focus of adult telemedicine. For instance, pediatric telemedicine emphasized parent training, child behavior management, and the continuity of early intervention, while adult telemedicine focused more on individualized disease management (33, 34). Through comparative analysis, it could be seen that pediatric telemedicine had shown significant advantages in meeting the special needs of children, but it also faced more technological, ethical, and policy challenges.

**Figure 8:**
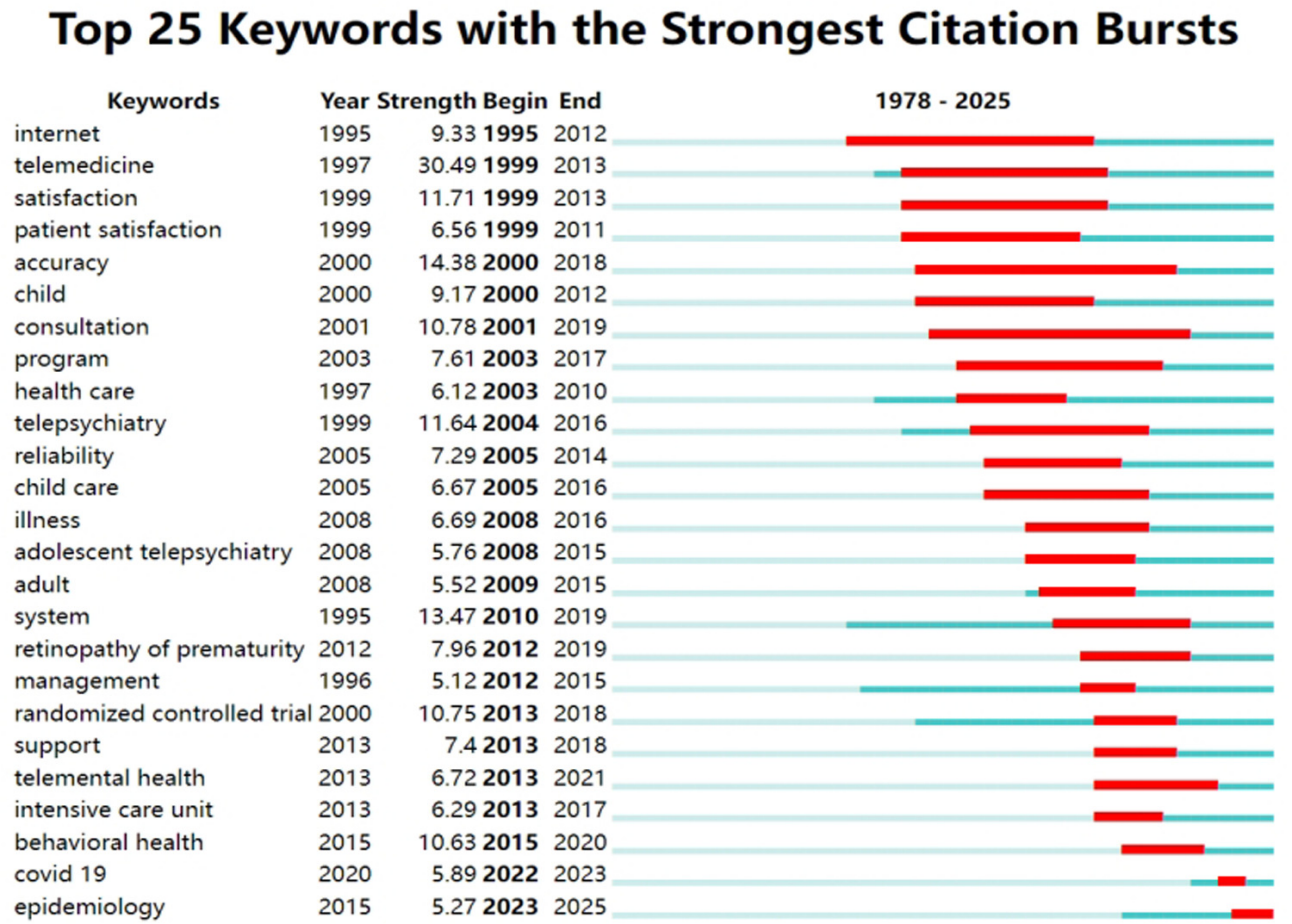
Keywords bursting map for research on pediatric telemedicine.

To flag transformative trends, analyzing keywords bursting allowed researchers to identify past research frontiers and emerging trends, highlighting the most dynamic and potentially transformative areas. The top 25 keywords with citation bursts spanned from 1978 to 2025 (Figure 8). Year indicated the initial year of the keyword's appearance. Begin and End denoted the keyword's start and end years with citation bursts, while the length of the red line corresponded to the duration of the keyword burst. In the field of children's telemedicine, the burst for the keywords “accuracy” and “consultation” lasted the longest, approximately 20 years. This indicated that researchers had been consistently concerned about the accuracy of telemedicine throughout the course of children's telemedicine research, and the long-term role of telemedicine was to consult on health issues.

The keyword “COVID-19” lasted only one year, possibly due to the convenience of telemedicine technology brought to the attention of medical personnel. Telemedicine reduced the need for pediatric patients to travel to hospitals, thereby decreasing the likelihood of cross-infection during the new coronavirus pneumonia pandemic. The epidemiology trends had persisted until the time of analysis. Telemedicine could break geographical limitations, allowing medical resources (such as experts, technology, etc.) to be more widely distributed and utilized. The application of telemedicine in pediatric epidemiology also reflected the continuous progress of medical technology. With the rapid development of Internet technology, communication technology, artificial intelligence, and other technologies, telemedicine had become increasingly powerful and more reliable.

## 4 Limitations

There are two limitations to this study. Firstly, due to language limitations, non-English article was not included. Secondly, the use of CiteSpace software for data analysis resulted in the collection of solely relevant literature from the WoSCC database. Consequently, future studies should involve the analysis of various literature databases to reduce potential bias.

## 5 Conclusion

This study analyzed articles from the field of pediatric telemedicine research up to the present day. A quantitative and visual review of findings and advances in the field was conducted using CiteSpace analysis software. Basic information, such as the number of annual publications, authors, institutions, countries, and journals, was quantitatively analysed. The number of publications has grown rapidly in recent years, with an exponential increase. The rapid development of telemedicine, facilitated by the new coronavirus pandemic, has led to significant growth in the field of pediatric medicine. Quantitative analysis indicates that the majority of papers, as well as the top 10 institutions and authors, are from the United States, highlighting the country's important position in the field of pediatric telemedicine. The main research points and development directions in the field of pediatric telemedicine for each period can be identified by analysing the keywords. Finally, this analysis focuses on the emergence of keywords during the development of telemedicine technology in the field of pediatrics over the past 20 years. Pediatric scholars have continuously summarised and innovated in terms of the quality of tele-diagnosis and care, the accuracy of telemedicine disease diagnosis, as well as telemedicine's impact on children's mental and behavioural health. This has led to the development and application of telemedicine technology in the field of pediatrics.

Although telemedicine technology has shown promise in pediatrics, a retrospective analysis has identified some limitations. (1) It is important to note that telemedicine cannot replace face-to-face physician examination and diagnosis. Face-to-face communication is necessary to establish trust between doctor and patient. (2) While telemedicine can manage certain diseases, it is important to note that complex medical problems involving major decisions, such as infections in organ transplant recipients or rejection reactions, require in-person evaluation by a clinician. (3) Although telemedicine has proven successful in emergency care, any patient presenting with a progressive acute illness should be evaluated in person by a qualified medical professional. Therefore, we recommend that the initial patient visit should be a face-to-face encounter between the physician and the patient. For patients with stable conditions, telemedicine follow-up can be used for monitoring. We need to strike a balance between the convenience of telemedicine and the benefits of face-to-face contact.

Future research should explore strategies for effectively integrating telemedicine into existing pediatric healthcare delivery models. This includes investigating the impact of telemedicine on workflows, provider roles, and interdisciplinary collaboration, developing models for seamless transitions between telemedicine and in-person care, and conducting cost-benefit analyses across different settings. There is an urgent need for standardized protocols for telemedicine across various pediatric subspecialties. This includes establishing clear guidelines for patient selection, assessment, interventions, and follow-up. Furthermore, it is necessary to evaluate the sustained impact of telemedicine on children's health outcomes, including long-term clinical status, quality of life, and parental satisfaction.
